# Supplementary material for: Targeted vaccination is effective for mpox clade Ib in England despite increased household transmission: Predictions from a modelling study
Source: PLOS Glob Public Health. 2025 Oct 8;5(10):e0005188. doi: 10.1371/journal.pgph.0005188 (PMC12507221; doi:10.1371/journal.pgph.0005188)
Supplement: Attachment — Submitted filename: ResponsetoReviewers.docx [file pgph.0005188.s002.docx]

**Reviewers’ reports**

**Comments to the Author**

1. Does this manuscript meet PLOS Global Public Health’s [publication criteria](https://track.editorialmanager.com/CL0/https:%2F%2Fjournals.plos.org%2Fglobalpublichealth%2Fs%2Fcriteria-for-publication/1/010f019872ed624b-5e09be50-e216-4870-b315-0092634d16f2-000000/a25xvHLlXDDHOWD_Zj2aGBTSgPkFTKkNtkPmtufuDbU=222)? Is the manuscript technically sound, and do the data support the conclusions? The manuscript must describe methodologically and ethically rigorous research with conclusions that are appropriately drawn based on the data presented.

Reviewer #1: Yes

Reviewer #2: Yes

2. Has the statistical analysis been performed appropriately and rigorously?

Reviewer #1: Yes

Reviewer #2: Yes

3. Have the authors made all data underlying the findings in their manuscript fully available (please refer to the Data Availability Statement at the start of the manuscript PDF file)?

The [PLOS Data policy](https://track.editorialmanager.com/CL0/https:%2F%2Fjournals.plos.org%2Fglobalpublichealth%2Fs%2Fdata-availability/1/010f019872ed624b-5e09be50-e216-4870-b315-0092634d16f2-000000/ftszhkZ90GTzb1X6ZpQFaASi95TCqbs6LVI2kj_kciA=222) requires authors to make all data underlying the findings described in their manuscript fully available without restriction, with rare exception. The data should be provided as part of the manuscript or its supporting information, or deposited to a public repository. For example, in addition to summary statistics, the data points behind means, medians and variance measures should be available. If there are restrictions on publicly sharing data—e.g. participant privacy or use of data from a third party—those must be specified.

Reviewer #1: Yes

Reviewer #2: Yes

4. Is the manuscript presented in an intelligible fashion and written in standard English?

PLOS Global Public Health does not copyedit accepted manuscripts, so the language in submitted articles must be clear, correct, and unambiguous. Any typographical or grammatical errors should be corrected at revision, so please note any specific errors here.

Reviewer #1: Yes

Reviewer #2: Yes

5. Review Comments to the Author

Please use the space provided to explain your answers to the questions above. You may also include additional comments for the author, including concerns about dual publication, research ethics, or publication ethics. (Please upload your review as an attachment if it exceeds 20,000 characters)

*~~Reviewer #1: 2. Statistical analysis appropriateness and rigor
The statistical methods employed appear broadly appropriate for a secondary analysis of cross-sectional survey data. The use of logistic regression to assess associations between sociodemographic factors and access to modern contraceptive methods is methodologically sound. However, I recommend the following improvements:

Specify the rationale for the selection of variables included in the adjusted models.

Clarify whether multicollinearity diagnostics or interaction terms were assessed.

Indicate whether complex survey design features—such as sampling weights, stratification, and clustering—were appropriately accounted for in the analyses.
The Data Availability Statement adequately identifies the DHS Program as the data source. To enhance transparency, I recommend:

Including a direct URL to the DHS dataset repository:~~* [*~~https://dhsprogram.com/data/available-datasets.cfm~~*](https://dhsprogram.com/data/available-datasets.cfm) *~~Indicating the exact dataset(s) used (e.g., GUIR7AFL.DTA) and whether syntax files for analysis are available upon request or as supplementary material.~~*

*~~4. Language and clarity of presentation
The manuscript is generally intelligible and coherent. Nonetheless, several improvements in scientific writing are advised:

Divide overly long paragraphs to improve readability and information retention.

Revise complex sentences with multiple subordinate clauses to enhance clarity.

Ensure consistent use of tense, especially in the Results and Methods sections.

Address minor grammatical issues and punctuation inconsistencies throughout the text.

General Comments to the Author
The Introduction section provides valuable background but could be more concise, with a sharper focus on the specific gaps in access to contraception among indigenous women.

The Discussion section would benefit from broader comparisons with similar studies conducted in other Latin American indigenous populations.

Consider expanding the policy implications and programmatic relevance of the findings in the Guatemalan context, as well as in comparable global health settings.

Methodological limitations are appropriately acknowledged, though the absence of qualitative insights could be mentioned as a constraint in capturing sociocultural determinants.~~*

The comments from reviewer 1 relate to a different manuscript. We informed the editorial office of this error.

Reviewer #2: The manuscript presents a well-structured modeling framework to estimate the reproduction number of mpox (clade Ib) in England and compares targeted versus non-targeted vaccination strategies. The approach is methodologically reproducible, I was able to implement some models to verify the statements. The results provide a rationale for prioritizing individuals with higher contact rates in vaccination strategies.

Many thanks for your positive review, and for checking the reproducibility of our framework through implementing models.

To improve clarity, reproducibility, and usefulness for public health applications, I suggest the following minor revisions:
1. Reproducibility – GitHub Repository
The code is useful, but the repository would benefit from a few practical additions:Processed Data Tables, Include de-identified, aggregated tables (e.g., weighted reproduction numbers, contact type distributions) derived from the original datasets. These should allow replication of the main results without disclosing individual-level data.

Thanks for these comments:

a) Under the NATSAL end user license, we cannot upload processed data tables, however we have included code for processing the data, and a description of obtaining the data, which are freely available upon registration with the UK Data Service. The Persistent identifier (DOI) is: <https://doi.org/10.5255/UKDA-SN-7799-2>. It should be straightforward to anyone to register and access the data. We have added the DOI to the data availability section of the manuscript, and included detail in the README file on github.
b) We have cleaned up the github repository to include a README file, instructions for running the code <https://github.com/bristol-vaccine-centre/MPox-Reckoners>.

2. Interpretation and Limitations
A few limitations could be better contextualized in the Discussion section:
-Data Sources:Clarify that the NATSAL-3 datasets are subject to: Self-reporting bias, especially for stigmatized behaviors; Underrepresentation of high-risk MSM populations or those less likely to be sampled in household surveys.

We have added details on representativeness and biases to paragraph 3 in the discussion:

*“In this study, we used data from the National Surveys of Sexual Attitudes and Lifestyles 3 (NATSAL3) to inform the transmission model. NATSAL provides valuable sexual behavioural data, spanning multiple years. All surveys, including NATSAL, have variable representativeness and reporting biases, which may particularly affect some questions on sensitive topics. Due to sampling by postcodes, NATSAL does not include people who are homeless or living in institutions, however measured biases have remained largely consistent across surveys”*

-Generalizability: Briefly mention that the findings rely on contact and household structures typical of the UK. In other contexts, variations in household composition or sexual network structures could affect both transmission patterns and intervention outcomes.

We agree and have added a statement about generalisability to the fourth paragraph in the discussion:

*“Our results are dependent on the contact and household structure in the UK, therefore are not necessarily applicable to other settings. In particular, the lack of association between household size and number of sexual contacts (i.e. individuals reporting high numbers of sexual contacts reported small household sizes), may not apply elsewhere.”*

3. Future Directions
It would be helpful to briefly expand the Conclusions or Discussion to note future rounds of behavioral surveys (e.g., NATSAL-4) could include more detailed household and sexual contact modules. Integration with clinical surveillance systems could also improve estimates of secondary attack rates and vaccine effectiveness in practice.

We completely agree. In the third paragraph of the discussion, we have expanded the text to say:

*“Here, the inclusion of household size data, in addition to sexual behaviour, enabled us to capture non-sexual close contact transmission in the model; in future, inclusion of social contact data as part of NATSAL would further enhance the power of models […] The integration of clinical data would allow direct calculation of secondary attack rates and vaccine effectiveness, rather than relying on external estimates.”*

These adjustments will improve the manuscript’s clarity and usability without changing its core results. The conclusion—that targeted vaccination is more efficient, even under increased household transmission—is well-supported. With these updates, the work will be more practical for informing future modeling and public health strategies.
